# Metabolic Adaptation to Following Temperature Acclimation in Fish on the Yun-Gui Plateau

**DOI:** 10.3390/biology14121645

**Published:** 2025-11-22

**Authors:** Wei Dao, Jing Long, Yanhui Yin, Anli Wu, Yuanchao Chen, Haitao Gao, Xiaofu Pan, Junxing Yang, Xiaoai Wang, Yuanwei Zhang

**Affiliations:** 1 State Key Laboratory of Genetic Evolution & Animal Models, Kunming Institute of Zoology, Chinese Academy of Sciences, Kunming 650201, China; daowei01@163.com (W.D.); longjing995@163.com (J.L.); yinyanhui603@163.com (Y.Y.); wuanli@mail.kiz.ac.cn (A.W.); 15187602713@163.com (Y.C.); xiaofupan@163.com (X.P.); yangjx@mail.kiz.ac.cn (J.Y.); 2 Yunnan Key Laboratory of Plateau Fish Breeding, Kunming Institute of Zoology, Chinese Academy of Sciences, Kunming 650201, China; 3 University of Chinese Academy of Sciences, Beijing 100049, China; 4 Yunnan Institute of Fishery Sciences Research, Kunming 650111, China; ynkhtt@126.com

**Keywords:** metabolic rate, temperature, high altitude, adaptation strategies

## Abstract

Temperature is crucial for fish physiology, as metabolism and related physiological processes are directly influenced by thermal energy. Against the backdrop of global warming, climate-induced variations in water temperature increasingly constrain fish physiology. Consequently, understanding the effects of temperature on fish metabolism is vital for predicting how global warming might impact various fish species. Plateau fish, predominantly cold-water species, exhibit greater sensitivity to temperature fluctuations. However, research on the effects of temperature on plateau fish is currently limited. Consequently, this study employed low-altitude *H. nobilis* as a reference while *D. macrophthalmus* and *A. grahami* were selected from the Yun–Gui Plateau. Following 15 days of temperature acclimation at 10, 15, 20, 25, and 30 °C, organ mass, the resting metabolic rate, and mitochondrial function were measured. The results indicated that high-altitude fish exhibit heightened metabolic sensitivity, demonstrating more pronounced increases or decreases in metabolic rates as temperature increases, along with limited plasticity in organ size. This may render high-altitude fish more vulnerable to the impacts of climate warming. Furthermore, physiological differences between altitudes and species were observed, primarily characterized by higher metabolic rates across all measured temperatures in plateau species. Additionally, plateau fish presented greater masses of heart, red muscle, and liver but smaller masses of brain and kidney. We propose that the trade-off between elevated metabolic rates and organ size may represent an adaptive strategy for fish inhabiting high-altitude environments, involving specific ecological costs and benefits. These findings not only address the knowledge gap regarding the metabolic characteristics of fish on the Yun–Gui Plateau but also provide theoretical and experimental foundations for the conservation of high-altitude fish populations.

## Simple Summary

The study depicted the metabolic responses of plateau fish to temperature changes. Plateau fish exhibit higher metabolic sensitivity, with more pronounced changes in metabolic rates as temperature increases, coupled with limited organ size plasticity, making them potentially more vulnerable to climate warming. Additionally, these fish demonstrate higher metabolic rates and larger heart, red muscle, and liver masses. The trade-off between high metabolic rates and organ size may represent an adaptive strategy to high-altitude environments. This research fills a knowledge gap regarding the metabolic characteristics of fish on the Yun-Gui Plateau.

## 1. Introduction

In the context of global warming, increasing attention has been given to understanding organismal responses to ongoing climate change, with considerable research focused on examining variations in thermal tolerance and acclimation both within and among species [1]. The metabolic rate is frequently one of the earliest traits to respond to climate change and is considered a key ecological parameter [2,3]. Among ectotherms, temperature is one of the most critical and extensively studied factors influencing metabolic rates [4]. Generally, the metabolic rate increases with temperature; however, this increase is bounded by a thermal limit, beyond which the metabolic rate decreases with increasing temperature [5,6,7,8]. Consequently, the metabolic rate reflects evolutionary optimization within species, making understanding metabolic patterns crucial to contemporary ecological theory [4,9]. Metabolism also varies among species according to latitude, altitude, and climate zone [10]. For example, the metabolic cold adaptation (MCA) hypothesis suggests that organisms from cold environments (high latitudes or altitudes) exhibit higher metabolic rates than those from warmer regions when measured at identical temperatures [4]. This phenomenon may serve as a mechanism to sustain physiological function under cold conditions [11,12]. Additionally, the climate variability (CVH) hypothesis posits that species from stable environments tend to have narrower temperature ranges, facilitating evolutionary specialization [13,14] and consequently exhibiting lower physiological plasticity [15,16]. Conversely, species from more variable habitats demonstrate broader thermal tolerance and greater thermal plasticity, allowing rapid adaptation to highly variable climates [3,16,17]. Such species also maintain relatively lower metabolic rates over wider temperature ranges to minimize energy expenditure [13]. Therefore, assessing how metabolic rates respond to temperature fluctuations is crucial for evaluating the vulnerability of species to climate change.

Fish represent a significant group of aquatic vertebrates and display extensive diversity across altitudes and climatic zones. Their growth [18], reproduction [19,20], and metabolism [21,22,23] are strongly influenced by temperature. The metabolic rate, which represents kinetic energy expenditure at the cellular level, is a fundamental requirement for fish growth and survival and is especially susceptible to temperature changes. Organisms can adaptively modulate their metabolic responses to temperature through phenotypic plasticity or genetic evolution [24,25]. Phenotypic plasticity may involve alterations at the organ, individual, or cellular/subcellular level. In particular, the metabolic rate can be modified by adjusting the organ size (e.g., liver, brain, and heart) and mitochondrial function [26,27,28]. Mitochondria act as cellular energy ‘powerhouses’, providing approximately 90% of ATP through oxidative phosphorylation (OXPHOS), thus underpinning nearly all animal activities [8,29,30]. For example, acute warming initially triggers an increase in OXPHOS, enabling tissues to meet increased metabolic demands and sustain ATP production [7]. However, mitochondrial capacity becomes compromised (plateauing or declining) near a fish’s upper thermal limit [6,7,8]. Currently, water temperature shifts driven by climate change increasingly constrain the physiological functions of fish [31]. Plateau fish, predominantly cold-water species, have garnered significant attention because of their increased sensitivity to climate change. The Yun–Gui Plateau is located in the low-latitude transitional region east of the Qinghai–Tibet Plateau and is one of China’s four major plateaus [32]. Through natural evolution and historical development, this region has gradually produced numerous highly specialized endemic cold-water fish species [33,34]. However, previous research on fish from the Yun–Gui Plateau has focused predominantly on conservation and systematic evolution, whereas studies on fish from other plateau regions have also concentrated on conservation [35,36,37] and genetic foundations [38,39]. These genetic studies indicate that adaptation to high altitudes involves genetic modifications related to energy metabolism, yet evidence for phenotypic plasticity adjustments in plateau fishes remains limited. Consequently, it is imperative to investigate metabolic adaptations in plateau fishes at the organ, individual, and mitochondrial levels following temperature changes.

**Figure 1:**
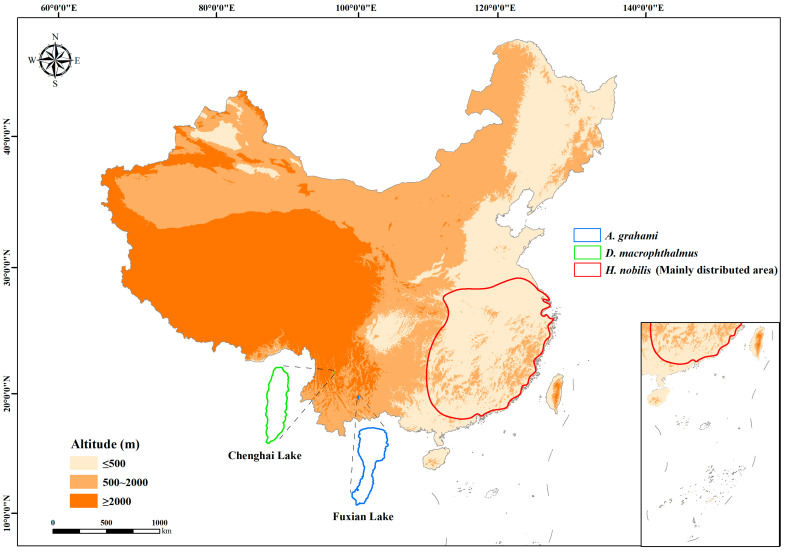
Distribution areas of *H. nobilis*, *D. macrophthalmus*, and *A. grahami*.

To elucidate the metabolic responses of plateau fishes to temperature fluctuations and characterize their metabolism, we employed low-altitude *Hypophthalmichthys nobilis* as a reference species. We then conducted comparative analyses of the metabolic responses to temperature changes in two plateau species, *Distoecodon macrophthalmus* and *Anabarilius grahami*. *H. nobilis* is widely distributed at low altitudes (average altitude < 500 m), primarily in subtropical and temperate zones (e.g., the Yangtze River, Pearl River, and their affiliated lakes in central and southern China), with a natural habitat temperature range of 4–34 °C [40]. *D*. *macrophthalmus* and *A*. *grahami* are cold-water species endemic to lakes on the Yun-Gui Plateau. Both species are economically important and have long adapted to low-temperature environments; thus, they are considered narrow-temperature-range species. *D. macrophthalmus* exclusively inhabits Chenghai Lake, Yunnan (altitude: 1503 m), with a natural habitat temperature range of 9–28 °C [41,42]. *A. grahami* exclusively inhabits Fuxian Lake, Yunnan (altitude: 1721 m), with a natural habitat temperature range of 12–23 °C (Figure 1) [41,43]. All three species belong to the family Xenocyprididae (Cypriniformes) [44] and are omnivorous, primarily feeding on plankton. In this study, individuals of each species were subjected to temperature treatments (10, 15, 20, 25, and 30 °C) for 15 days, after which organ sizes, resting metabolic rates, and mitochondrial respiration rates were measured. By analyzing variations in organ size, the resting metabolic rate (RMR), and mitochondrial function across these three fish species, we obtained valuable insights into the metabolic characteristics of plateau fishes. Our findings may provide novel perspectives for elucidating the physiological adaptation mechanisms of fish inhabiting high-altitude environments [45,46].

## 2. Materials and Methods

All experimental fish (juvenile *H. nobilis*, *D. macrophthalmus*, and *A. grahami*) were obtained from the Endangered Fish Conservation Center (EFCC) of the Kunming Institute of Zoology (KIZ), Chinese Academy of Sciences, Kunming, Yunnan, China.

### 2.1. Thermal Acclimation

Before the experiments, the fish were acclimated for two weeks in a recirculating-water tank system. During acclimation, the water temperature was maintained at 20 ± 0.5 °C, and the dissolved oxygen (DO) concentration was maintained above 5 mg/L. Commercial feed (composed of crude protein 36.0%, crude fat 6.0%, crude fiber 6.0%, crude ash 12.0%, calcium 0.5–1.5%, total phosphorus 0.9%, sodium chloride 0.3–1.6%, lysine 2.2%, and moisture 12.5%) was provided daily to satiate between 17:00 and 18:00. Approximately one-third of the water volume was replaced daily to prevent ammonia nitrogen accumulation. The photoperiod was set to 12 h light and 12 h dark.

After body weight (BW) and standard body length (SL) were measured, the fish of each species were randomly assigned to warm or cold acclimation treatments. BW content was similar among the *H. nobilis* (BW: 21.33 ± 4.74 g, SL: 9.66 ± 1.26 cm), *D. macrophthalmus* (BW: 23.84 ± 6.83 g, SL: 10.93 ± 0.86 cm), and *A. grahami* (BW: 20.58 ± 2.93 g, SL: 12.16 ± 0.66 cm) treatments. Each treatment group was gradually adjusted to the experimental temperature (10 °C, 15 °C, 25 °C, and 30 °C) at a rate of 1 °C per day and then maintained for 15 days. After acclimation, the RMR, organ size, and mitochondrial respiration rate (liver, brain, heart, kidney, and red muscle) were measured. During temperature acclimation, the fish were fed, the water quality was maintained, and the photoperiod remained consistent with the adaptive acclimation phase. The fish were fasted for 48 h before the measurements were taken to ensure complete evacuation of the gastrointestinal contents [47].

### 2.2. Resting Metabolic Rate Measurement

After acclimation, the RMR of each fish was determined via a flow-through respirometer (laboratory self-designed) at five temperatures (10, 15, 20, 25, and 30 °C), and the measurement method followed Yan et al. [47] and Fu et al. [48]. The fish were then individually placed in the respirometer chamber and allowed to acclimate for approximately 12 h before the experiment. The next day, oxygen consumption was measured ten times at 1 h intervals, and the means were used as the RMRs. Each respirometry chamber housed one fish, with one additional chamber left empty as a blank control. The water temperature was controlled within ±0.5 °C. The water flow rates were adjusted before the measurements were taken to ensure that the DO concentrations in the chambers remained above 70% saturation to avoid hypoxic stress. This adjustment maintained a DO difference of 1.0–2.0 mg L^−1^ between the experimental and blank control groups.

### 2.3. Organ Size Determination

After the RMR measurements were completed at all temperatures, three fish from each treatment of each species were sacrificed and dissected for organ size determination. The fresh livers, brains, hearts, kidneys, and red muscles were removed, and the samples were weighed (0.0001 g) immediately. Then, the mitochondria of fresh tissues were extracted following published methods for measuring mitochondrial respiration and citrate synthase (CS) activity [47,49]. The organ index for the livers, hearts, brains, kidneys, and red muscles was calculated via the following formula: Organ Index (%) = (organ mass/body mass) × 100%.

### 2.4. Mitochondrial Isolation

After the fish were euthanized, heart, liver, kidney, brain, and red muscle tissues were immediately collected and weighed. Hearts, brains, and kidneys, as well as approximately 0.1–0.2 g each of liver and red muscle tissue, were collected. The tissue samples were coarsely chopped with scissors, suspended in 1.5 mL of isolation buffer A (250 mM sucrose, 10 mM TES, 1 mM EDTA, 0.4% BSA, pH 7.4), and homogenized via a glass–Teflon homogenizer. The homogenates were centrifuged at 12,100× *g* for 10 min to remove lipid droplets. The pellet, containing cell debris, nuclei, and mitochondria, was resuspended in 1 mL of ice-cold isolation buffer B (250 mM sucrose, 10 mM TES, 1 mM EGTA, 0.4% BSA, pH 7.4) and centrifuged again at 980× *g* for 10 min to remove nuclei and cellular debris. The supernatants were transferred to fresh tubes and centrifuged at 8800× *g* for 10 min. The mitochondrial pellets were resuspended (1:1, *w*/*v*) in ice-cold isolation buffer C (100 mM KCl, 20 mM TES, and 1 mM EGTA, pH 7.4). All procedures were conducted on ice.

Mitochondrial respiration levels in various tissues were measured at 10, 15, 20, 25 and 30 °C using a temperature-controlled Clark electrode system (Hansatech Instruments, Pentney, UK). A 100-μL mitochondrial suspension was added to the assay medium (1.25 mM succinate, 1.25 mM pyruvate, 1.25 mM glutamate, 0.75 mM malate, 50 mM Tris-base, 1 mM EDTA, 15 mM KH_2_PO_4_, 5 mM MgCl_2_·6H_2_O, 250 mM sucrose, pH 7.4) to reach a final volume of 2 mL. The maximal OXPHOS rate (state III respiration) was measured after adding ADP to a final concentration of 0.25 mM.

The mitochondrial protein content was determined via the Folin phenol method, with bovine serum albumin used as a standard. The mitochondrial respiration rates were calculated as nmol O_2_ min^−1^ mg^−1^ mitochondrial protein. The respiratory control ratio (RCR) was calculated as the ratio of state III respiration to state IV or state II respiration to determine the coupling rates.

### 2.5. CS Activity Measurement

After the fish were euthanized, heart, liver, kidney, brain, and red muscle tissues were immediately collected, weighed, immersed in liquid nitrogen, and stored at −80 °C. CS activity was measured via a Fish Citrate Synthase (CS) ELISA Kit (Beijing Jinzhiyan Biotechnology Co., Ltd., Beijing, China). The specific method and steps for enzyme extraction followed the kit instructions. The absorbance (OD value) was measured at 450 nm via a microplate reader at 20 °C, and the enzyme activity was calculated according to the provided formula. Protein concentrations in homogenates were measured using bovine serum albumin as a standard. Enzyme activities are expressed as U mg^−1^ protein. CS is a critical enzyme in aerobic respiration, and previous studies have shown a strong correlation between CS activity and mitochondrial content in tissues. Therefore, this study used tissue CS activity as an indicator of the mitochondrial content instead of the mitochondrial protein content [50].

### 2.6. Temperature Quotient Calculations (Q_10_)

The *Q*_10_ value is an indicator of metabolic sensitivity to temperature changes. We calculated the thermal sensitivity (*Q*_10_) of metabolic rates and mitochondrial respiration rates as follows:

$$Q_{10}={\left(\frac{M_{2}}{M_{1}}\right)}^{\left(\frac{10}{T_{2-T_{1}}}\right)}$$

where *M*_2_ is the biological rate (e.g., state III respiration) at temperature *T*_2_ (e.g., 20 °C) and where *M*_1_ is the rate at temperature *T*_1_ (e.g., 25 °C). Typically, the *Q*_10_ values range between 2 and 3, indicating that the reaction rate doubles or triples with a 10 °C temperature change [45,51,52].

### 2.7. Statistical Analysis

All the data were analyzed via SPSS 27.0 (SPSS Inc., Chicago, IL, USA). Before analysis, normality and homogeneity of variance were tested. One-way ANOVA followed by post hoc Tukey’s tests was used to analyze the mitochondrial respiration rates, mitochondrial protein content, and CS activity among the tissues of the three species. Two-way ANOVA was used to assess potential interactions between experimental variables (temperature, species, and tissue) and mitochondrial respiration or the mitochondrial content (Table A1). Three-way ANOVA was applied to evaluate the effects of temperature, tissue, and species on mitochondrial respiration rates and mitochondrial content (Table A2). All values are presented as the means ± standard errors (SEs). *p* < 0.05 was considered statistically significant.

## 3. Results

### 3.1. Organ Sizes

**Figure 2:**
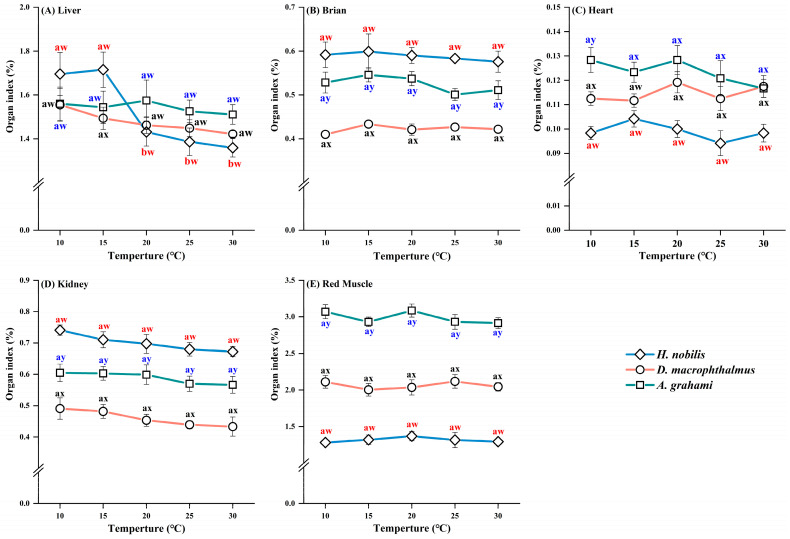
Organ indices of *H. nobilis*, *D. macrophthalmus*, and *A. grahami* tissues. The data are expressed as the means ± SEs, *n* = 12; a and b indicate significant differences between temperatures (*p* < 0.05); w, x, and y indicate significant differences between species (*p* < 0.05).

The brain, heart, kidney, and red muscle indices did not differ significantly between treatments in any species (Table A1). However, the liver index in *H. nobilis* was approximately 1.2-fold greater under cold treatments (10 and 15 °C) than under warm treatments (25 and 30 °C), whereas the liver indices did not differ between the treatments in *D. macrophthalmus* and *A. grahami* (Figure 2). Organ indices differed significantly among the five tissues across species. The heart and red muscle indices of *D. macrophthalmus* and *A. grahami* were greater than those of *H. nobilis*, whereas the brain and kidney indices showed the opposite pattern. The liver index of *H. nobilis* was greater than that of *D. macrophthalmus* and *A. grahami* at 10 and 15 °C but lower at 20–30 °C (Figure 2).

### 3.2. Resting Metabolic Rate (RMR)

**Figure 3:**
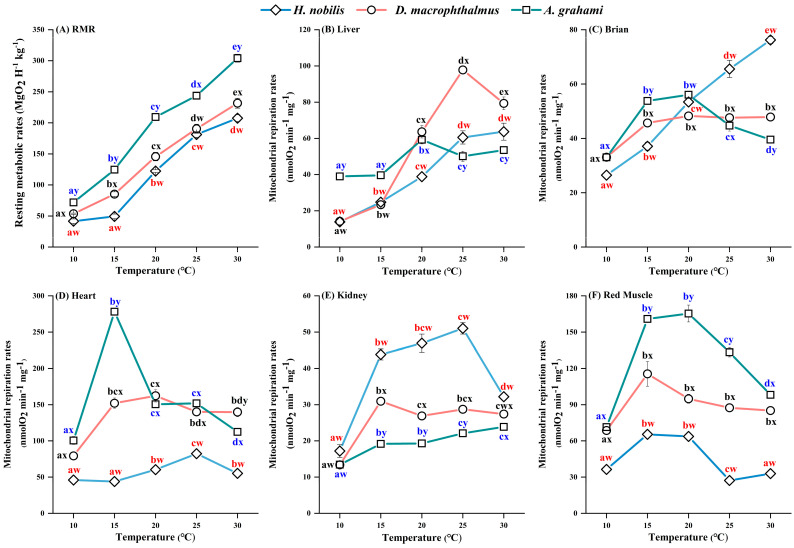
Effects of temperature on the RMRs and mitochondrial respiration rates of *H. nobilis*, *D. macrophthalmus*, and *A. grahami*. (**A**): the resting metabolic rates, *n* = 6; (**B**–**F**): the mitochondrial respiration rates in the tissues, *n* = 4. The data are presented as the means ± SEs; a–e indicate significant differences between temperatures (*p* < 0.05); w–y indicate significant differences between species (*p* < 0.05).

For all three species, the RMR increased with temperature (10–30 °C) (Figure 3A). The RMRs were different between the temperature treatments in *D. macrophthalmus* and *A. grahami* but not between the 10 °C and 15 °C treatments in *H. nobilis*. At all the acclimation temperatures, *A. grahami* had the highest RMR, followed by *D. macrophthalmus*, with *H. nobilis* having the lowest (Figure 3A).

### 3.3. Mitochondrial Respiration Rates and the RCR

In all the species, the mitochondrial respiration rates of all the tissues initially increased but subsequently decreased with increasing temperature, but the temperature at which peak respiration occurred varied among the species and tissues (Figure 3B–F). In *H. nobilis*, the liver and brain mitochondria increased consistently with temperature, whereas the heart and kidney rates peaked at 25 °C, decreased at 30 °C, and the red muscle rates plateaued at 15 °C. In *D. macrophthalmus*, liver mitochondria peaked at 25 °C and then declined at 30 °C, whereas other tissues plateaued or peaked at 15 °C. In *A. grahami*, liver mitochondria peaked at 20 °C and then declined at 25 °C, with other tissues plateauing or peaking at 15 °C. Among the species, heart tissue presented the highest mitochondrial respiration rates, followed by red muscle, brain, and liver, with the kidney showing the lowest rates (Figure 3B–F). At all temperatures, the mitochondrial respiration rates of heart and red muscle tissues from *D. macrophthalmus* and *A. grahami* were significantly greater than those of *H. nobilis* (Figure 3D,F), whereas kidney mitochondria presented the opposite pattern (Figure 3E). Under cold treatments (10 and 15 °C), liver and brain mitochondrial respiration rates were higher in *D. macrophthalmus* and *A. grahami* than in *H. nobilis* (Figure 3B,C). However, under warm conditions (25 and 30 °C), brain mitochondrial respiration was higher in *H. nobilis* (Figure 3C).

Temperature significantly affected the RCR values (Table A1). The RCR values for most tissues of the three species were greater than 3 at most temperatures, first increasing but then decreasing as the temperature increased (Figure 3B–F). In *H. nobilis*, the kidney RCR was only 1.96 at 10 °C, and red muscle had lower RCR values (1.32–2.51) across temperatures (Table 1). Notably, at 30 °C, the RCR values of most tissues in the three species were low (<3) (Table 1), indicating a reduced coupling rates of mitochondrial respiration at relatively high temperatures.

### 3.4. Mitochondrial Protein Content and CS Activity

**Figure 4:**
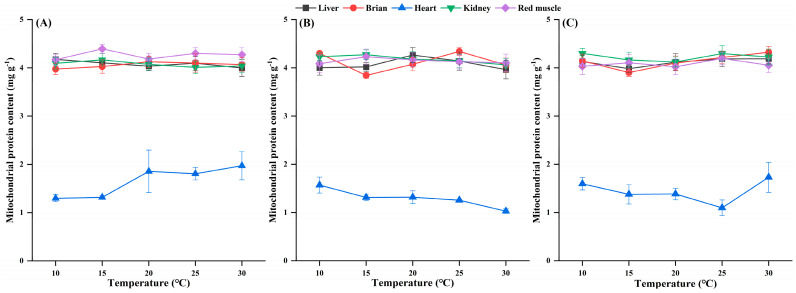
Effects of temperature on the mitochondrial protein content in the tissues of *H. nobilis* (**A**), *D. macrophthalmus* (**B**), and *A. grahami* (**C**). The data are expressed as the means ± SEs, *n* = 4.

Under each temperature treatment, the mitochondrial protein content did not differ significantly among the three species (Table A1 and Table A2). The heart mitochondrial protein content was significantly lower than that in other tissues but not different from that in other tissues (Figure 4).

**Figure 5:**
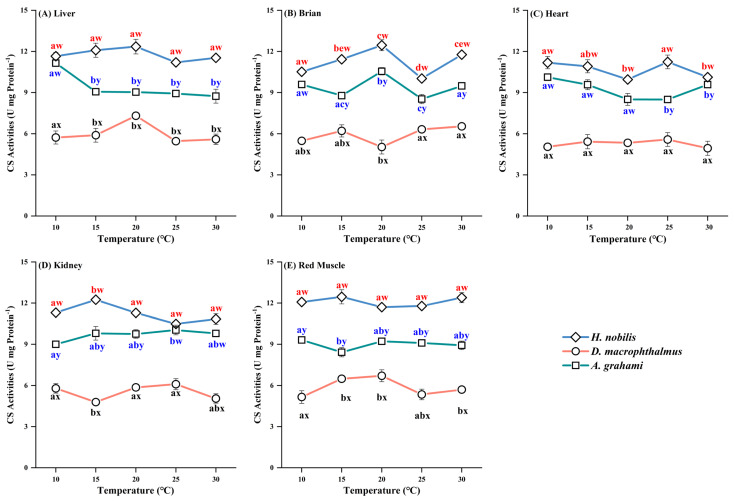
Effects of temperature on CS activity in the tissues of *H. nobilis*, *D. macrophthalmus*, and *A. grahami*. The data are expressed as the means ± SEs, *n* = 3; a–e indicate significant differences between temperatures *(p* < 0.05); w–y indicate significant differences between species (*p* < 0.05).

Significant differences in CS activity were observed among species across the temperature treatments (Table A1 and Table A2). Among all the tissues, *H. nobilis* had the highest CS activity, followed by *A. grahami*, with *D. macrophthalmus* exhibiting the lowest activity. CS activity in *D. macrophthalmus* was the lowest. The CS activity of *D. macrophthalmus* was significantly lower than that of the other two species, whereas the differences between *H. nobilis* and *A. grahami* were not significant (Figure 5).

### 3.5. Thermal Sensitivity (Q_10_)

The three species presented considerable variability in terms of their *Q*_10_ values across the experimental temperature range (Table 2). The *Q*_10_ values indicated greater thermal sensitivity at lower temperatures (10–15 °C and 15–20 °C) and decreased at higher temperatures (20–25 °C and 25–30 °C) (Figure 4). Overall, high-altitude species presented greater thermal sensitivity in terms of metabolic rates at both lower (10–15 °C) and higher (25–30 °C) temperatures than did low-altitude species.

## 4. Discussion

### 4.1. Comparison of Metabolic Rates

Temperature is a crucial factor influencing the metabolic requirements of fish. Typically, metabolic rates initially increase with increasing temperature, reach a peak, and subsequently decline [13]. In this study, the resting metabolic rates (RMRs) of all three species increased with temperature (Figure 3A), whereas the mitochondrial respiration rates in the tissues first increased but then decreased (Figure 3B–F). In general, mitochondrial metabolic capacity is regulated by alterations in mitochondrial abundance and/or oxidative capacity [48]. CS activity, which reflects flux rates in the tricarboxylic acid cycle and is often used as a proxy for mitochondrial density, shows a pattern inconsistent with mitochondrial respiration rates [53]. Specifically, CS activity remained relatively stable across acclimation conditions when acute temperatures matched acclimation temperatures, suggesting enzyme quantity and/or quality adjustments [53]. The adjustment of mitochondrial respiration rates to changing temperatures is likely primarily mediated by modifications in mitochondrial oxidative capacity. Extensive research has demonstrated that temperature significantly influences metabolic rates in ectotherms. We observed that the *Q*_10_ values for all three species presented marked metabolic stress responses at relatively low temperatures, which decreased with increasing temperature, which was consistent with observations in other taxa [54]. Beyond 30 °C, respiratory rates decline (*Q*_10_ < 1.0), indicating impaired mitochondrial function [54]. Although temperatures exceeding 30 °C were not investigated in this study, metabolic efficiencies across all species contributed to declining *Q*_10_ values with increasing temperature, resulting in lower *Q*_10_ values at higher temperatures (Table 2). The RCR serves as a sensitive indicator of mitochondrial functional status [55]. Low RCR values indicate impaired mitochondrial ATP synthesis and dysfunction, whereas high RCR values reflect robust cellular activity and enhanced metabolism [56,57]. The RCR values exhibited a pattern similar to that of *Q*_10_, decreasing at higher temperatures (30 °C) (Table 1). These findings suggest that 30 °C may exceed the optimal physiological temperature range for these species. We also noted that this influence varies among species. Notably, high-altitude fish (*D. macrophthalmus* and *A. grahami*) exhibited steeper ascending and descending metabolic phases overall (Figure 3). This divergence likely reflects not only acute or passive temperature responses but also differences in thermophysiology, with high-altitude fish exhibiting greater metabolic thermosensitivity.

With respect to metabolic rates, our findings align with the MCA hypothesis. Specifically, high-altitude fish (*D. macrophthalmus* and *A. grahami*) presented greater metabolic rates than did low-altitude species (*H. nobilis*) across all measured temperatures (Figure 3). Such elevated metabolic rates may confer significant advantages for ectotherms, enabling faster food processing and providing organisms with enhanced cellular capacity to respond rapidly to environmental challenges [9,58]. At the mitochondrial level, we also observed greater energy allocation in the cardiac and red muscle tissues of high-altitude species, potentially reflecting an adaptation to prolonged low temperatures and resource scarcity in high-altitude environments [59,60]. The high metabolic capacity of cardiac and red muscle tissues enables plateau fish to consume oxygen more effectively, maintain blood flow, and satisfy the energy demands associated with extended food foraging periods [61]. Moreover, the elevated metabolic rates of plateau fish do not result from increased mitochondrial content but is instead achieved through comparatively fewer mitochondria, suggesting an efficient, resource-conserving strategy.

### 4.2. Changes in Organs

Organ size is among the most significant intrinsic factors influencing metabolic scaling [62]. Among various organs, the liver, and heart contribute most substantially to the whole-organism metabolic rate [6,27]. During thermal or seasonal acclimation, some fish species undergo adjustments in the relative mass of certain organ tissues to meet the energy metabolic demands under different temperature environments [63,64]. In this study, under cold acclimation (10 °C and 15 °C), the liver of *H. nobilis* was approximately 1.2 times larger than that under warm acclimation, indicating that cold compensation of the liver in *H. nobilis* may be achieved at the whole organ rather than at the tissue level [65]. From an ecological perspective, many physiological challenges posed by temperature fluctuations may be partially counterbalanced by adjustments in organ size [66]. In contrast, the brain, heart, kidney, and red muscle of *H. nobilis*, as well as organ sizes in high-altitude fish (*D. macrophthalmus* and *A. grahami*), showed no response to temperature acclimation, remaining relatively constant across all acclimation temperatures (Figure 2). The precise mechanisms remain unknown, though one hypothesis is that the short acclimation period limited the phenotypic plasticity of these organs. Similarly, the lack of change in liver size in plateau fish may stem from restricted plasticity, which partially supports CVH. However, owing to their limited plasticity, these organs may experience stronger selective pressures, thus promoting rapid adaptive evolution [67,68,69] and enabling unique local adaptations. We observed that high-altitude species (*D. macrophthalmus* and *A. grahami*) presented higher organ indices for the heart, red muscle, and liver than *H. nobilis* did (Figure 2A,C,E). The greater heart mass observed in plateau fish likely enhances blood circulation and transport capacity, serving as the “powerhouse” of the circulatory system [61], and may also improve resistance to extreme cold conditions [70] liver, the primary energy storage organ in fish [70], plays a crucial role in environmental adaptation. Additionally, red muscle continuously generates ATP through efficient aerobic metabolism (OXPHOS), thus supporting prolonged swimming activity [63]. Conversely, we found that the organ indices of the brain and kidney were lower in plateau fish (Figure 2). This finding likely arises from resource allocation constraints at high altitudes, where metabolic processes among organs may interact: some organs enlarge, whereas others shrink to maintain energy balance and ensure sustained growth [71]. This strategy may effectively conserve energy, allowing high-altitude species to maintain normal physiological functions.

### 4.3. Contribution of Mitochondria to Physiological Thermal Limits

Mitochondria provide cellular energy through OXPHOS and are essential for aerobic metabolism and maintaining the organismal energy balance. Consequently, temperature-induced impairments in mitochondrial function may critically affect thermal tolerance in animals [5,6]. Mitochondria have been proposed as key determinants of whole-organism thermal limits [72]. Moreover, mitochondrial physiological adjustments associated with thermal acclimation may lead to dysfunction during acute temperature changes, potentially resulting in shifts in thermal limits [5]. As temperature increases, mitochondrial oxygen consumption initially increases; however, beyond optimal physiological temperatures, oxygen consumption either plateaus or decreases, impairing ATP production [6,72,73]. Numerous studies suggest that high-temperature-induced declines in mitochondrial function significantly determine physiological thermal limits in many ectotherms [72]. Consistent with previous findings, the mitochondrial respiration rates in this study initially increased with increasing temperature but stabilized or declined at relatively high temperatures (Figure 3B–F), and the RCR values exhibited a similar trend (Figure 4). This finding suggests reduced coupling rates in mitochondrial OXPHOS, indicating that temperatures may exceed optimal physiological thresholds. Interestingly, among the five tissues examined, the heart was the first to show reduced mitochondrial function (Figure 3C), suggesting that the heart may be the earliest organ to fail under thermal stress. Numerous studies have also identified the heart as particularly vulnerable to heat stress, which restricts energy provision and consumption when fish exceed their optimal thermal limits [26,72,74,75,76,77]. Thus, we speculate that the temperature at which heart mitochondrial respiration peaks may represent a species’ optimal physiological temperature. In this study, the maximum heart respiration rates occurred at 25 °C (*H. nobilis*), 20 °C (*D. macrophthalmus*), and 15 °C (*A. grahami*), which aligns with their previously documented optimal growth temperatures [40,41,42,43,78]. The mitochondrial energy transduction efficiency of the electron transfer and phosphorylation system is often employed as an indicator of mitochondrial function and dysfunction [78]. While our investigation centered on the oxidative phosphorylation (OXPHOS) process, it did not include the electron transfer process itself. Additionally, other critical aspects—such as reactive oxygen species (ROS) production and the antioxidant defense system, mitochondrial membrane integrity (fluidity), permeability, and stability—have been strongly linked to animal thermal tolerance [72,73,79]. These aspects warrant further investigation in future research.

## 5. Conclusions

Currently, research on the metabolic responses and characteristics of plateau fishes during temperature acclimation is limited and remains unclear. This study revealed that the basal metabolic rates of all three species increased with increasing temperature, whereas the mitochondrial respiration rates across tissues initially increased but subsequently decreased. Under all the acclimation conditions, the CS activity remained relatively constant across the three species, indicating that changes in the mitochondrial respiration rate were independent of the mitochondrial content. In contrast, plateau fish species (*D. macrophthalmus* and *A. grahami*) presented greater temperature sensitivity and lower plasticity, suggesting increased vulnerability to climate warming. Furthermore, we observed increased metabolic rates in both *D. macrophthalmus* and *A. grahami* across all acclimation temperatures, supporting the MCA and CVH hypotheses. At the organ level, the liver, heart, and red muscle presented increased organ indices, whereas the brain and kidney presented decreased indices. This adaptive strategy involving elevated metabolic rates and adjustments in organ size may confer advantages for plateau fish in maintaining energy balance, with significant implications for survival. Nevertheless, this study has several limitations. First, this study focused on short-term thermal acclimation. While our results demonstrate significant physiological plasticity, long-term adaptation involves genetic changes. Future studies should thus employ long-term designs to fully assess the adaptive potential of these fish to challenges like climate change, and to determine if the observed responses are heritable and contribute to evolutionary fitness. Second, our investigation centered on mitochondrial oxidative phosphorylation but omitted other critical aspects like reactive oxygen species (ROS) production or membrane potential. Measuring these parameters in future work is essential. Elucidating these dynamics remains a key future goal, which will provide a more holistic view of mitochondrial physiology and deepen insights into the evolutionary and developmental dimensions of thermal adaptation in fish.

## Figures and Tables

**Table 1:** RCR values of mitochondria isolated from five tissues of *H. nobilis*, *D. macrophthalmus*, and *A. grahami* in the presence of different temperatures.

Tissue	Species	Temperature (°C)				
		10	15	20	25	30
Liver	*H. nobilis*	4.83 ± 0.09 ^ax^	6.32 ± 0.23 ^bw^	5.25 ± 0.34 ^aw^	4.94 ± 0.42 ^aw^	3.31 ± 0.12 ^cw^
	*D. macrophthalmus*	4.19 ± 0.11 ^aw^	5.81 ± 0.33 ^bw^	7.07 ± 0.31 ^cx^	5.23 ± 0.36 ^bdw^	4.96 ± 0.17 ^adx^
	*A. grahami*	9.40 ± 0.16 ^ax^	7.69 ± 0.46 ^bx^	7.36 ± 0.37 ^bx^	3.69 ± 0.27 ^cx^	3.64 ± 0.21 ^cw^
Brian	*H. nobilis*	5.30 ± 0.31 ^aw^	4.26 ± 0.34 ^aw^	6.01 ± 0.15 ^acw^	4.42 ± 0.37 ^bdw^	4.61 ± 0.10 ^adw^
	*D. macrophthalmus*	5.66 ± 0.42 ^aw^	4.80 ± 0.19 ^bw^	5.11 ± 0.35 ^bw^	4.04 ± 0.01 ^bw^	2.69 ± 0.09 ^cx^
	*A. grahami*	5.57 ± 0.19 ^aw^	5.13 ± 0.38 ^aw^	5.11 ± 0.18 ^aw^	3.34 ± 0.13 ^bx^	2.63 ± 0.09 ^cx^
Heart	*H. nobilis*	3.85 ± 0.29 ^aw^	7.02 ± 0.70 ^bw^	6.66 ± 0.30 ^bcw^	5.27 ± 0.60 ^cw^	4.35 ± 0.21 ^aw^
	*D. macrophthalmus*	4.72 ± 0.11 ^ax^	5.37 ± 0.00 ^abx^	5.97 ± 0.43 ^bw^	3.53 ± 0.21 ^cx^	3.65 ± 0.17 ^cw^
	*A. grahami*	3.14 ± 0.17 ^ay^	5.43 ± 0.31 ^bx^	4.47 ± 0.21 ^cx^	3.16 ± 0.31 ^ax^	2.03 ± 0.10 ^dx^
Kidney	*H. nobilis*	1.96 ± 0.18 ^aw^	5.55 ± 0.33 ^bw^	4.16 ± 0.08 ^cw^	4.52 ± 0.11 ^cw^	2.29 ± 0.10 ^aw^
	*D. macrophthalmus*	5.19 ± 0.39 ^ax^	5.49 ± 0.42 ^aw^	5.03 ± 0.22 ^bx^	4.74 ± 0.30 ^bw^	2.06 ± 0.19 ^cw^
	*A. grahami*	3.09 ± 0.14 ^aw^	4.55 ± 0.15 ^bw^	1.94 ± 0.34 ^cy^	1.83 ± 0.24 ^cx^	1.62 ± 0.08 ^cw^
Red muscle	*H. nobilis*	2.51 ± 0.20 ^aw^	2.12 ± 0.18 ^aw^	1.59 ± 0.11 ^bw^	1.32 ± 0.08 ^bw^	1.38 ± 0.08 ^bw^
	*D. macrophthalmus*	5.26 ± 0.08 ^ax^	3.33 ± 0.36 ^bx^	1.59 ± 0.10 ^cw^	2.16 ± 0.09 ^dew^	1.80 ± 0.10 ^cew^
	*A. grahami*	4.36 ± 0.13 ^ax^	3.68 ± 0.26 ^by^	3.22 ± 0.17 ^bcx^	3.78 ± 0.20 ^bdy^	3.86 ± 0.13 ^dx^

Note: The date were expressed as mean ± SEs, *n* = 4; a–e: different superscripts in the same line indicate significant differences between different temperature of the same species (*p* < 0.05); w–y: different superscripts in the same column indicate significant differences among different species in the same tissue (*p* < 0.05).

**Table 2:** *Q*_10_ values for RMRs and mitochondrial respiration rates in *H. nobilis*, *D. macrophthalmus*, and *A. grahami* for each temperature range after short-term temperature adaptation.

Parameter		Species	Temperature Ranges			
			10–15 °C	15–20 °C	20–25 °C	25–30 °C
Resting metabolic rates		*H. nobilis*	1.28 ± 0.08	5.28 ± 0.22	2.66 ± 0.18	1.31 ± 0.02
		*D. macrophthalmus*	2.52 ± 0.14	2.93 ± 0.13	1.74 ± 0.13	1.48 ± 0.03
		*A. grahami*	3.14 ± 0.30	2.84 ± 0.09	1.36 ± 0.03	1.56 ± 0.02
Mitochondrial respiration rates	Liver	*H. nobilis*	3.17 ± 0.28	2.52 ± 0.18	2.43 ± 0.13	1.11 ± 0.06
		*D. macrophthalmus*	2.74 ± 0.18	7.47 ± 0.57	2.40 ± 0.19	0.66 ± 0.04
		*A. grahami*	1.04 ± 0.05	2.24 ± 0.14	0.71 ± 0.03	1.17 ± 0.11
	Brian	*H. nobilis*	1.97 ± 0.09	2.09 ± 0.11	1.51 ± 0.12	1.37 ± 0.09
		*D. macrophthalmus*	1.91 ± 0.06	1.12 ± 0.04	0.97 ± 0.03	1.01 ± 0.04
		*A. grahami*	2.65 ± 0.06	1.09 ± 0.05	0.64 ± 0.03	0.79 ± 0.06
	Heart	*H. nobilis*	0.95 ± 0.13	1.89 ± 0.11	1.87 ± 0.07	0.45 ± 0.03
		*D. macrophthalmus*	3.70 ± 0.32	1.14 ± 0.05	0.75 ± 0.05	1.00 ± 0.03
		*A. grahami*	7.80 ± 0.66	0.29 ± 0.01	1.02 ± 0.02	0.55 ± 0.03
	Kidney	*H. nobilis*	6.84 ± 0.82	1.15 ± 0.07	1.20 ± 0.08	0.40 ± 0.01
		*D. macrophthalmus*	5.44 ± 0.36	0.75 ± 0.02	1.15 ± 0.04	0.92 ± 0.04
		*A. grahami*	2.03 ± 0.04	1.01 ± 0.02	1.31 ± 0.05	1.17 ± 0.07
	Red muscle	*H. nobilis*	3.23 ± 0.10	0.95 ± 0.04	0.18 ± 0.01	1.45 ± 0.06
		*D. macrophthalmus*	2.88 ± 0.46	0.70 ± 0.09	0.85 ± 0.04	0.95 ± 0.03
		*A. grahami*	5.08 ± 0.12	1.06 ± 0.09	0.65 ± 0.03	0.54 ± 0.01

Note: These values represent the magnitude of increase in a rate with a 10 °C increase in temperature, such that a value of two represents a doubling of the rate per 10 °C increase, values of one represent thermal independence, and values of 0.5 represent a halving of the rate per 10 °C increase, *n* = 4–6.

## Data Availability

The raw data supporting the conclusions of this article will be made available by the authors on request.

## Abbreviations

ADP	Adenosine diphosphate
ATP	Adenosine triphosphate
CVH	Climate variability hypothesis
MCA	Metabolic cold adaptation hypothesis
OXPHOS	Oxidative phosphorylation
RMR	Resting metabolic rate
CS	Citrate synthase activity
RCR	Respiratory control ratio

## Appendix A

**Table A1:** Summary of two-way ANOVA results for metabolic parameters in *H. nobilis*, *D. macrophthalmus*, and *A. grahami*.

Species	Parameter	Factors/Interaction		
		Temperature	Tissue	Temperature * Tissue
*H. nobilis*	RMR	F_4, 25_ = 350.081, *p* < 0.001	—	—
	Mitochondrial protein content	F_4, 95_ = 0.008, *p* = 1.00	F_4, 95_ = 243.549, *p* < 0.001	F_16, 75_ = 0.275, *p* = 0.997
	CS activity	F_4, 70_ = 3.772, *p* < 0.05	F_4, 70_ = 7.378, *p* < 0.001	F_14, 52_ = 2.332, *p* < 0.05
	Mitochondrial respiration rates	F_4, 95_ = 11.673, *p* < 0.001	F_4, 95_ = 4.411, *p* < 0.05	F_16, 75_ = 48.221, *p* < 0.001
	RCR	F_4, 95_ = 3.426, *p* < 0.05	F_4, 95_ = 36.648, *p* < 0.001	F_16, 75_ = 9.992, *p* < 0.05
	Organ index	F_4, 295_ = 0.291, *p* = 0.884	F_4, 295_ = 663.610, *p* < 0.001	F_16, 275_ = 2.402, *p* < 0.05
*D. macrophthalmus*	RMR	F_4, 25_ = 150.766, *p* < 0.001	—	—
	Mitochondrial protein content	F_4, 95_ = 0.046, *p* = 0.996	F_4, 95_ = 438.312, *p* < 0.001	F_16, 75_ = 0.861, *p* = 0.614
	CS activity	F_4, 70_ = 1.144, *p* = 0.343	F_4, 70_ = 2.313, *p* = 0.066	F_14, 52_ = 1.540, *p* = 0.130
	Mitochondrial respiration rates	F_4, 95_ = 2.807, *p* < 0.05	F_4, 95_ = 73.712, *p* < 0.001	F_16, 75_ = 24.034, *p* < 0.001
	RCR	F_4, 95_ = 8.544, *p* < 0.001	F_4, 795_ = 13.060, *p* < 0.001	F_16, 75_ = 15.418, *p* < 0.05
	Organ index	F_4, 295_ = 0.802, *p* = 0.525	F_4, 295_ = 900.219, *p* < 0.001	F_16,275_ = 13.553, *p* < 0.001
*A. grahami*	RMR	F_4, 25_ = 295.432, *p* < 0.001	—	—
	Mitochondrial protein content	F_4, 95_ = 0.062, *p* = 0.993	F_4, 95_ = 319.933, *p* < 0.001	F_16,75_ = 0.325, *p* = 0.993
	CS activity	F_4, 70_ = 2.364, *p* = 0.061	F_4, 70_ = 1.521, *p* = 0.205	F_14, 52_ = 5.040, *p* < 0.001
	Mitochondrial respiration rates	F_4, 95_ = 2.750, *p* < 0.05	F_4, 95_ = 60.670, *p* < 0.001	F_16, 75_ = 167.354, *p* < 0.001
	RCR	F_4, 95_ = 9.503, *p* < 0.001	F_4, 95_ = 17.926, *p* < 0.001	F_16, 75_ = 23.893, *p* < 0.05
	Organ index	F_4, 295_ = 0.682, *p* = 0.605	F_4, 295_ = 1259.665, *p* < 0.001	F_16, 275_ = 10.746, *p* < 0.001

**Table A2:** Summary of three-way ANOVA results for metabolic parameters.

Parameter	Factors/Interaction			
	Species	Species * Temperature	Species * Tissue	Species * Temperature * Tissue
RMR	F_2, 75_ = 240.611, *p* < 0.05	F_8, 75_ = 7.916, *p* < 0.05	—	—
Mitochondrial protein content	F_2, 297_ = 0.029, *p* = 0.971	F_8, 255_ = 0.805, *p* = 0.805	F_8, 255_ = 1.269, *p* = 0.260	F_32, 255_ = 0.343, *p* = 1.00
CS activity	F_2, 222_ = 893.394, *p* < 0.001	F_8, 156_ = 3.130, *p* < 0.05	F_8, 156_ = 4.354, *p* < 0.001	F_28, 156_ = 2.904, *p* < 0.001
Mitochondrial respiration rates	F_2, 297_ = 13.586, *p* < 0.001	F_8, 255_ = 76.907, *p* < 0.001	F_8, 255_ = 455.965, *p* <0.001	F_32, 255_ = 53.852, *p* < 0.001
RCR	F_2, 297_ = 0.425, *p* = 0.654	F_8, 255_ = 17.233, *p* < 0.05	F_8, 255_ = 59.754, *p* < 0.05	F_32, 255_ = 11.859, *p* < 0.05
Organ index	F_2, 897_ = 8.164, *p* < 0.001	F_8, 825_ = 21.540, *p* < 0.0001	F_8, 825_ = 268.301, *p* < 0.001	F_32, 825_ = 12.667, *p* < 0.001
